# Association between caregiver health literacy and patient characteristics in pediatric new onset diabetes mellitus

**DOI:** 10.1016/j.puhe.2025.105892

**Published:** 2025-08-08

**Authors:** Jean Potter, Olivia Sterns, Sara Einis, Svetlana Azova, Caroline Kohler, Gretchen Waldman, Katharine Garvey, Erinn T. Rhodes

**Affiliations:** a Department of Nursing, Inpatient Medical Programs, Boston Children’s Hospital, Boston, MA, USA; b Division of Endocrinology, Boston Children’s Hospital, Boston, MA, USA; c Department of Pediatrics, Harvard Medical School, Boston, MA, USA; d Division of Gastroenterology, Hepatology, and Nutrition, Boston Children’s Hospital, Boston, MA, USA

**Keywords:** Health literacy, Diabetes mellitus, Pediatric, Length of stay, Continuous glucose monitor

## Abstract

**Objectives:**

This study aimed to describe the health literacy of caregivers of patients with new onset diabetes mellitus (NODM) presenting to a large, pediatric medical center and to describe the association between caregiver health literacy and patient characteristics.

**Study design:**

This was a retrospective, cohort study. Caregiver health literacy was assessed using the Newest Vital Sign (NVS). NVS is scored 0–6: adequate literacy (AL) (4–6); possibility of limited literacy (PLL) (2–3); and high likelihood of limited literacy (HLLL) (0–1).

**Methods:**

Bivariate and multivariable analyses were performed with caregiver health literacy as the predictor. P < 0.05 was considered significant.

**Results:**

Between January 1, 2016 and December 31, 2023, there were 1832 NODM patients [mean age 10.2 (4.8) years, 46 % female]; 1701 (92.8 %) caregivers completed NVS: 92.3 % AL, 5.2 % PLL, and 2.5 % HLLL. Among 1091 diagnosed before January 1, 2023, 561 (51.4 %) and 136 (12.5 %) utilized a continuous glucose monitor (CGM) and insulin pump, respectively, within 12 months. Lower health literacy was associated with higher odds of interpreter use (PLL p < 0.001; HLLL p < 0.001), of tailored diabetes education (PLL p < 0.001; HLLL p < 0.001), and longer length of stay (PLL p < 0.001; HLLL p < 0.001). HLLL (adjusted odds ratio [aOR] 0.25, 95 % confidence interval [CI] 0.06, 0.94) but not PLL (aOR 1.06, 95% CI 0.44, 2.57) was associated with lower odds of CGM use within 12 months.

**Conclusions:**

Patients with NODM whose caregivers have lower health literacy represent a high-risk population associated with increased resource utilization, longer hospitalization, and risk for decreased uptake of diabetes technology. A health-literacy informed approach to initial education may offer opportunities to support the individual needs of caregivers.

## 1. Introduction

There is an increasing incidence of type 1 diabetes mellitus (T1DM) and type 2 diabetes mellitus (T2DM) in youth in the United States (US) with an annual estimated incidence in 2017–2018 of 18,200 and 5,300, respectively.^1,2^ Diabetes mellitus (DM) is a complex condition requiring intensive self-management to achieve optimal outcomes, and while evolving diabetes technology has facilitated some aspects of diabetes management for patients and families,^3^ equitable access to diabetes therapies remains a concern.^4^ Addressing health literacy and implementing health-literate practices are recommended healthcare policies to enhance patient and family understanding of health information, reduce health disparities, and improve health outcomes.^5–8^

Based on the National Assessment of Adult Literacy in 2003, 36 % of US adults had basic or below-basic health literacy skills.^9^ Health literacy, as proposed in Healthy People 2030, addresses both personal health literacy and organizational health literacy.^10^ Personal health literacy is “the degree to which individuals have the ability to find, understand, and use information and services to inform health-related decisions and actions for themselves and others.”^10^ Organizational health literacy is “the degree to which organizations equitably enable individuals to find, understand, and use information and services to inform health-related decisions and actions for themselves and others.”^10^ Given the complexity of diabetes care and self-management skills required, the health literacy of caregivers of children with diabetes is an important consideration as it can have a significant impact on the caregivers’ ability to manage the health care needs of the child.^6,11–15^

Caregivers with limited health literacy may have difficulty understanding and applying health information, which can negatively impact the care and outcomes of youth.^6^ For those with new onset diabetes mellitus (NODM), this may lead to poor glycemic outcomes and increased risks for acute and long-term health complications.^6,16^ These risks may include hyperglycemia, diabetic ketoacidosis (DKA), or hypoglycemia related to caregiver medication errors with insulin dosing and/or improper decision-making and actions related to sick day management or preparation for moderate-to-vigorous physical activity or exercise.^6,13,17^ Prior studies of the association of caregiver health literacy and needs and outcomes in pediatric diabetes have focused on the outpatient setting and on youth with established diabetes.^18–21^ In several studies, lower caregiver health literacy has been associated with both differing communication needs for diabetes education^19^ and worse glycemic outcomes.^18,20^

The Health Literate Care Model supports patient and family engagement in health care by providing a framework of care for organizational health literacy that adopts a universal precautions approach.^22^ This approach acknowledges that all patients benefit from clear communication and that health literacy has multidimensional determinants, including ones that may be situational.^22,23^ However, limitations in health literacy can create a learning barrier in diabetes self-management education (DSME), and early assessment provides the healthcare team with the ability to modify diabetes education interventions if limitations are identified.^17,24,25^ Utilizing tailored, evidence-based, health-literate education approaches in the care of pediatric patients with chronic health conditions, such as DM, can support improved quality of health care, patient safety, and patient health outcomes.^26^

Through the implementation of a formal health literacy assessment at admission, the Inpatient Diabetes Program at Boston Children’s Hospital (BCH) established methods to tailor NODM education to address caregiver health literacy. This study aimed to describe the health literacy of caregivers of patients with NODM presenting to a large, pediatric tertiary care academic medical center from 2016 to 2023 and to examine the association of caregiver health literacy with patient sociodemographic, clinical, and diabetes management characteristics including uptake of diabetes technology within 12 months of diagnosis. We hypothesized that patients with NODM whose caregivers have lower health literacy have a more severe presentation, longer hospitalization, and receive additional instructional strategies during initial admission. In addition, families with lower health literacy are less likely to adopt diabetes technology within 12 months following diagnosis.

## 2. Methods

### 2.1. Study design and population

This was a retrospective, cohort study. The Inpatient Diabetes Program at BCH began assessing health literacy as part of the NODM admission process with the Newest Vital Sign (NVS)^27^ to inform clinical care in 2016. The study cohort included all patients presenting to BCH for admission with NODM requiring insulin from January 1, 2016 through December 31, 2023. Follow-up data up to 12 months post-diagnosis were collected for patients in this population diagnosed before January 1, 2023 and followed for outpatient care at BCH. Patients who had at least two outpatient medical appointments [doctor or diabetes nurse educator (DNE)] in that 12-month period were included.

### 2.2. Health literacy assessment and management

The BCH Inpatient Diabetes Program performs a health literacy assessment for caregivers of patients with NODM using the NVS in English or Spanish. The NVS is a valid and reliable tool which assesses numeracy, document literacy, and prose literacy, as opposed to only assessing reading level achievement.^27,28^ NVS consists of an ice cream container nutrition label and six assessment questions. Caregivers must interpret the information from the label to answer the questions. The tool offers a quick screening, approximately 3 minutes, to identify limitations in health literacy and has been used to assess caregiver health literacy in the pediatric setting.^29–32^ NVS is scored on a scale of 0–6 with standardized categories for adequate literacy (AL) (4–6), possibility of limited literacy (PLL) (2–3), and high likelihood of limited literacy (HLLL) (0–1).^27,28^ For caregivers with NVS scores of 4 or less or with languages other than English or Spanish, who could not complete the NVS, the DNE completes additional individualized assessments. These assessments along with the NVS inform development of an individualized DSME plan based on the caregiver’s cultural beliefs, language, learning style, health literacy level, and support systems. When appropriate, tailored diabetes education resources, such as visual or pictorial education tools, simplified insulin regimens, or individualized daily schedule/plan, are utilized in addition to standard written and spoken communication methods for NODM education (Supplementary Fig. 1).

### 2.3. Data collection

Sociodemographic and clinical data were obtained from the electronic medical record and from inpatient and outpatient diabetes quality improvement databases. The latter include tracking for completion and scoring of NVS, methods used for diabetes education, presence of DKA on presentation, and utilization of diabetes technology including continuous glucose monitor (CGM) and insulin pump (IP). The database did not document who completed the NVS. In most cases, the caregiver was a parent/guardian although a patient (≥18 years) may have completed an assessment. If multiple individuals were assessed, the lowest score was documented, and this focused the education and management. Sensitivity analysis was performed to confirm results were not impacted by including all patients.

### 2.4. Statistical analysis

Descriptive data are presented as mean (standard deviation [SD]), median (interquartile range [IQR]) or proportions, as appropriate. Bivariate analyses were evaluated with Chi-square, ANOVA, and Kruskal-Wallis tests, as appropriate. Sociodemographic characteristics included patient age, sex, insurance, and race and ethnicity. Clinical characteristics included presence of DKA and hemoglobin A1c (HbA1c) at presentation. Diabetes management was characterized by need for interpreter services, use of tailored diabetes education at diagnosis, and length of stay (LOS), as well as use of diabetes technology (CGM and IP) within 12 months post-diagnosis. Multivariable logistic regression was used to evaluate the association of caregiver health literacy with DKA, use of tailored diabetes education, use of interpreter services, and use of diabetes technology. For the diabetes technology models, a sensitivity analysis included only patients diagnosed between 2019 and 2022, as uptake increased in 2019. Models were adjusted for sociodemographic characteristics and, for diabetes technology, also adjusted for LOS, use of tailored diabetes education, DKA, and diagnosis year. Multivariable linear regression was used to evaluate the association of caregiver health literacy with HbA1c adjusted for sociodemographic variables and DKA. Generalized linear model with Gamma log link regression was used to evaluate the association of caregiver health literacy with LOS adjusted for sociodemographic variables, DKA, and tailored diabetes education. Race and ethnicity were not included in multivariable models due to unknown data (6 %) and collinearity with insurance (p < 0.001). P < 0.05 was considered significant. Analyses were performed with SPSS (Version 29.0.0.0, Chicago, IL).

## 3. Results

### 3.1. Description of study population

Between January 1, 2016 and December 31, 2023, there were 1832 patients admitted with NODM requiring insulin initiation. The majority (92 %) had T1DM and spoke either English (93 %) or Spanish (3 %). Most patients were white, non-Hispanic (65 %), Hispanic (11 %), or black, non-Hispanic (8 %). Mean age was 10.2 (4.8) years, and 46 % were female. Additional characteristics are shown in Table 1. Bivariate relationships (Table 1) were similar when patients ≥18 years were excluded.

### 3.2. Caregiver health literacy and patient demographic and clinical characteristics

Of the 1832 patients with NODM, a total of 1701 (92.8 %) caregivers completed the NVS. Of these, there were 92.3 % with AL, 5.2 % with PLL, and 2.5 % with HLLL. Among those with incomplete NVS (n = 131), the majority occurred in 2016–2017 (63 %) during initial implementation of screening or if caregivers spoke a language other than English or Spanish (20 %). In bivariate analyses (Table 1), lower caregiver health literacy was associated with public health insurance (p < 0.001), greater use of an interpreter (p < 0.001), greater use of tailored diabetes education methods (p < 0.001), higher prevalence of DKA (p = 0.01), higher HbA1c (p = 0.03), and longer LOS (p < 0.001). After adjusting for sociodemographic characteristics, lower caregiver health literacy remained significantly associated with higher odds of interpreter use and use of tailored diabetes education although the association with DKA was attenuated (Table 2). Attenuation of the relationship with DKA was primarily due to age (p < 0.001) and insurance (p < 0.001) with younger age and public insurance associated with higher odds of DKA.

A multivariable regression model evaluating the association of caregiver health literacy and HbA1c was significant (F = 46.72, p < 0.001), after adjustment for sociodemographic variables and DKA, although only explained 15.3 % of the variance. A significant association with HbA1c remained only for caregivers with PLL (β = 0.048, p = 0.046) but not with HLLL (β = 0.02, p = 0.41). When PLL and HLLL were combined, the association with HbA1c was also significant(β = 0.05, p = 0.041) although the effect size remained small. LOS remained significantly longer for patients with NODM whose caregivers had lower health literacy even when adjusted for sociodemographic characteristics, DKA, and use of tailored diabetes education [PLL Exp(B) 1.5 (95 % confidence interval [CI] 1.3, 1.6), p < 0.001; HLLL Exp(B) 1.6 (95 % CI 1.4, 1.9), p < 0.001].

### 3.3. Association of caregiver health literacy with uptake of diabetes technology

**Fig. 1.**
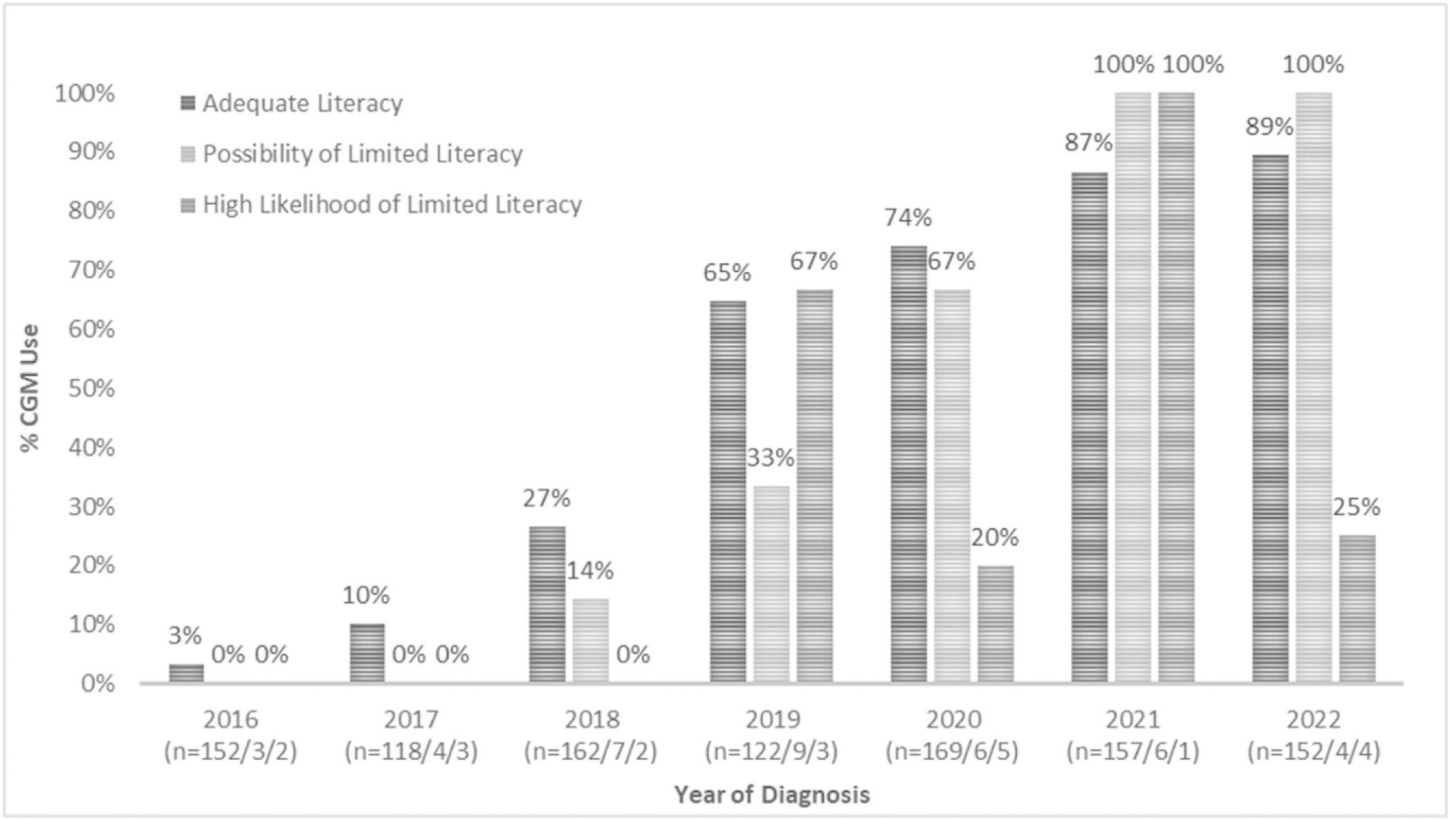
Percent of patients using continuous glucose monitor (CGM) within 12 months of diagnosis by year of diagnosis and stratified by caregiver health literacy. N in parentheses represents number of caregivers in given year with adequate, possibility of limited, and high likelihood of limited health literacy for whom patient CGM use data were available.

**Fig. 2.**
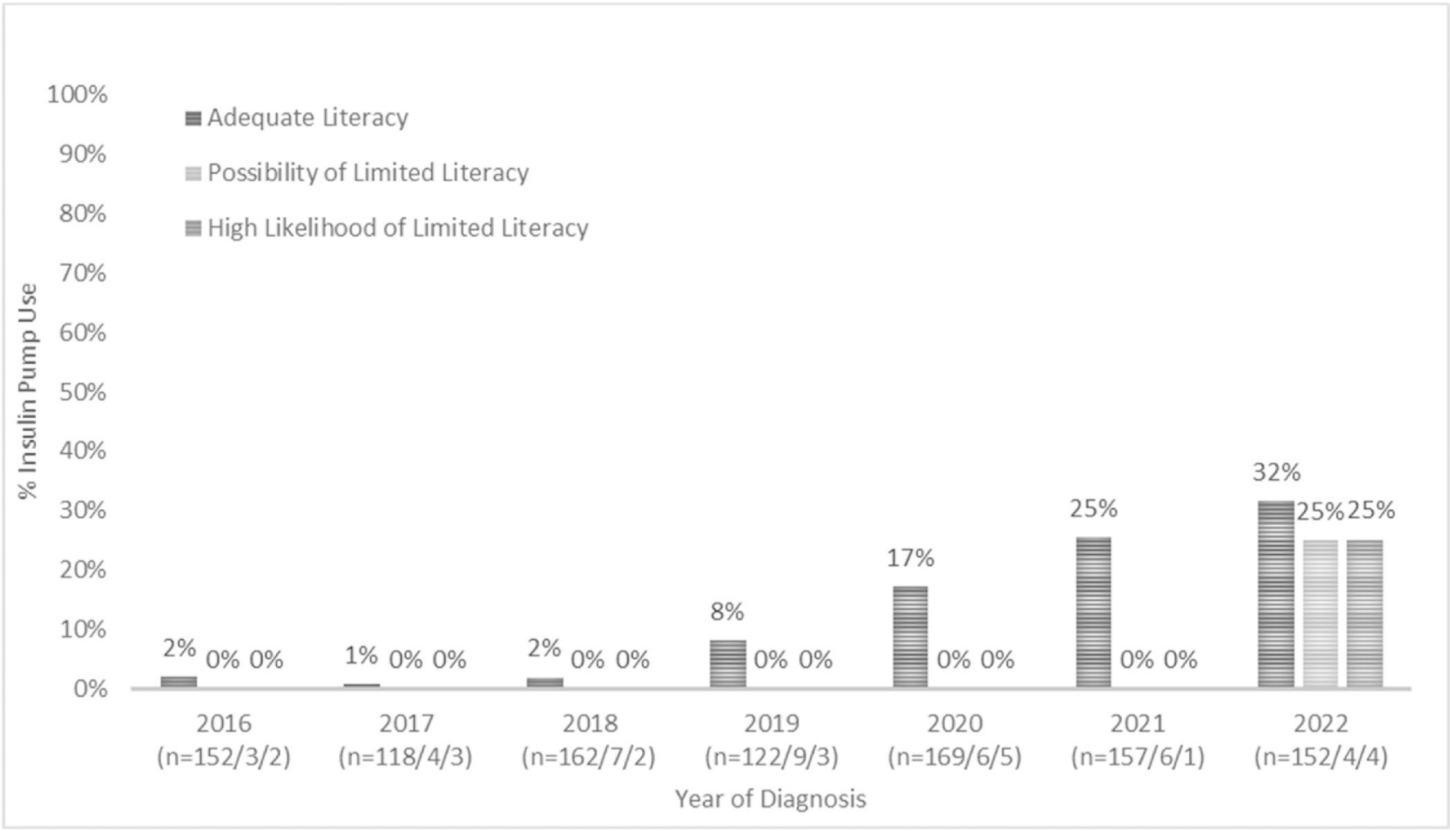
Percent of patients using an insulin pump within 12 months of diagnosis by year of diagnosis and stratified by caregiver health literacy. N in parentheses represents number of caregivers in given year with adequate, possibility of limited, and high likelihood of limited health literacy for whom patient insulin pump use data were available.

Among the 1204 patients who followed up at BCH for outpatient care, 1091 (90.6 %) had available data on CGM and IP use within 12 months of diagnosis. Of these, 561 (51.4 %) and 136 (12.5 %) had utilized CGM and IP, respectively. Uptake of diabetes technology changed significantly during the time period analyzed (2016–2023) with 3.2 % of those diagnosed in 2016 using CGM within 12 months compared to 88.1 % of those diagnosed in 2022 (p < 0.001). Similarly, 1.9 % of those diagnosed in 2016 used IP within 12 months compared to 31.1 % in 2022 (p < 0.001). Among caregivers with AL and PLL, 67 % or more had children using CGM within 12 months beginning in 2020 (Fig. 1). IP were not used by children of caregivers with PLL or HLLL until 2022 (Fig. 2).

In unadjusted analyses across the whole sample (Table 3), HLLL was associated with lower odds of CGM use within 12 months (odds ratio [OR] 0.31, 95 % CI 0.11, 0.86). After adjustment, HLLL remained associated with lower odds of CGM use within 12 months (aOR 0.25, 95 % CI 0.06, 0.94) with similar findings when limited to diagnosis between 2019 and 2022 (Table 3). An interaction between caregiver health literacy and diagnosis year was not significant.

The relationship between caregiver health literacy and IP use was not significant (Table 3). The most significant predictors of IP use included later diagnosis year (p < 0.001) and private insurance (p < 0.001) (data not shown).

## 4. Discussion

Patients with NODM whose caregivers have lower health literacy represent a high-risk population.^13^ We found that 7.7 % of caregivers of patients with NODM had lower health literacy, and lower caregiver health literacy was associated with use of additional resources, including interpreters and tailored diabetes education, and significant extension of the LOS. Further, children of caregivers with HLLL were significantly less likely to use CGM within 12 months of diagnosis.

Prior studies of caregiver health literacy in the context of pediatric diabetes have focused on the outpatient setting in youth with established diabetes.^18–21,33,34^ In these studies, lower caregiver health literacy or numeracy has been associated with worse glycemic outcomes in some,^18,20^ but not all,^21,33^ studies. Although studies have not all utilized the same screening methods, Hassan et al. utilized the NVS with 200 caregivers of children with established T1DM and reported that mean HbA1c in children whose caregivers had inadequate literacy (HLLL) was significantly higher than in those with AL (p < 0.001).^18^ We hypothesized that patients whose caregivers had lower health literacy would have a more severe presentation of diabetes. Although this was supported by bivariate analyses with an association between lower caregiver health literacy and greater likelihood of DKA and higher HbA1c at presentation, these relationships were attenuated in adjusted analyses, suggesting that a new presentation with pediatric diabetes may have a more complex overall set of biopsychosocial determinants.^35^

Hospitalization for a new diagnosis of diabetes can be a stressful event for families, reinforcing the concept that health literacy can be a dynamic, situational state requiring a universal precautions approach.^22,36^ Therefore, in the setting of NODM, an interprofessional care approach with use of clear written and spoken communication and teach-back methods to assess comprehension is recommended for all in the delivery of DSME.^13,16,24,36,37^ Early assessment of health literacy can provide the health care team with the ability to further modify diabetes education interventions if specific limitations are identified.^17,24,25^ This study adds new information about caregiver health literacy in the setting of pediatric diabetes, focusing on the inpatient setting for youth with NODM. The need to consider health literacy in child health has garnered increasing attention,^6,13,36^ and the pediatric inpatient setting has been identified as having opportunities for evaluation and improvement.^11,32^

Consistent with our hypotheses, lower caregiver health literacy was associated with longer LOS even after adjustment for sociodemographic, clinical, and diabetes management characteristics. Prior pediatric studies have demonstrated an association between lower health literacy and greater healthcare utilization, including emergency department (ED) visits and hospitalization, particularly for children with asthma and medical complexity.^11,36,38^ For patients admitted for NODM and their caregivers, new information is presented requiring a range of health literacy skills, including numeracy.^15,39^ Health-literacy informed DSME interventions may enhance caregiver and patient knowledge and self-care abilities and improve downstream glycemic and health outcomes.^11,16,17,24^ There may also be opportunities for quality improvement ultimately leading to decreased hospital LOS, reduced ED visits, decreased readmissions to the hospital, reduced healthcare costs, increased support, and increased patient, family and medical provider satisfaction.^6,11,13,36^

Consistent with our hypothesis, patients whose caregivers had HLLL were significantly less likely to use CGM within 12 months of diagnosis. This relationship persisted when adjusted for sociodemographic characteristics and for the trend in overall increased uptake of CGM use over the 7-year period evaluated. CGM use has increased significantly over the last decade in the US and around the world.^3^ However, disparities in the uptake of diabetes technology have been observed.^4,40^ In the Type 1 Diabetes Exchange registry in the US, evaluation of data from 2016 to 2018 demonstrated the use of diabetes technology is lowest and HbA1c is highest in those in the lowest socioeconomic status quintile.^40^ A recent study including data from this and other registries suggests that early use of diabetes technology may also afford benefits in longer term glycemic outcomes, even among those presenting with more severe diabetes.^41^ Our findings reinforce that there are still opportunities for improvement^4^ and support addressing caregiver health literacy in the broader context of social determinants of health.^26^

Several limitations of the analysis should be noted. We conducted this initiative at a single institution, limiting the generalizability of our findings. Our insulin-requiring NODM population primarily has T1DM, and most patients had caregivers with adequate health literacy. While patients, aged 18 years or older, may have completed an assessment, the education and management supported all health literacy needs of the family as reflected by the documented NVS score, and our sensitivity analysis confirmed that our results were not impacted by including all patients. Future work focusing on patients diagnosed with T2DM, including in the outpatient setting, may identify additional associations. Further, as the number of caregivers with PLL or HLLL was small, we may not have had adequate power to identify significant associations with some outcomes, including diabetes technology, although the observed association for CGM remained significant when secular trends in technology were considered. Future work with a longer timeline following diagnosis may offer additional opportunity to assess diabetes technology and glycemic outcomes.^40^ With regard to language, we were only able to conduct the formal NVS assessment for English- and Spanish-speaking caregivers, although this represented the majority (96 %) of our clinical population. However, the needs of patients speaking other languages do warrant additional attention^42^ particularly given the observed overlap between interpreter use and lower health literacy, and this will inform future approaches. The NVS has been widely used in the clinical setting.^27,30,43,44^ However, future work may consider whether alternatives^31,45,46^ including diabetes-specific tools, such as the Diabetes Numeracy Test,^15^ may be helpful along with the interventions that target diabetes-specific health literacy.^47^ Finally, we chose to focus on caregiver health literacy in the inpatient setting as this was felt to be the most relevant for NODM education. However, in the outpatient setting, additional focus on adolescents may be useful as patients take on additional responsibilities for their own diabetes management.^39^

This study evaluated the impact of the integration of caregiver health literacy assessment into the inpatient diabetes management for youth with NODM. Overall, we found patients whose caregivers had lower health literacy were a high-risk group that utilized greater resources; were hospitalized longer; and for those with HLLL, were significantly less likely to utilize a CGM within 12 months. Utilizing a health-literacy informed approach to initial diabetes education is important for all patients and families,^5^ and completing health literacy assessments with caregivers prior to initiating diabetes education may offer additional opportunities to adapt education strategies to meet the individual needs of caregivers.^24^

## Supplementary Material

MMC1

Appendix A. Supplementary data

Supplementary data to this article can be found online at https://doi.org/10.1016/j.puhe.2025.105892.

## Figures and Tables

**Table 1:** Association of patient demographic and clinical characteristics with caregiver health literacy.

	Overall N = 1832		Adequate Literacy n = 1570		Possibility of Limited Literacy n = 88		High Likelihood of Limited Literacy n = 43		P-value^a^
	n	n (%) or mean (SD)	n	n (%) or mean (SD)	n	n (%) or mean (SD)	n	n (%) or mean (SD)	
**Sociodemographic**									
Patient Age, years	1832	10.2 (4.8)	1570	10.1 (4.8)	88	10.3 (4.9)	43	10.5 (4.7)	0.85
Female sex	1832	843 (46.0 %)	1570	713 (45.4 %)	88	35 (39.8 %)	43	17 (39.5 %)	0.45
Insurance	1832		1570		88		43		<0.001
Public		621 (33.9 %)		458 (29.3 %)		65 (73.9 %)		40 (93.0 %)	
Private		1202 (65.6 %)		1104 (70.3 %)		23 (26.1 %)		3 (7.0 %)	
Other^b^		9 (0.5 %)		8 (0.5 %)		0 (0.0 %)		0 (0.0 %)	
**Clinical and Diabetes Management**									
DKA	1832	699 (38.2 %)	1570	584 (37.2 %)	88	44 (50.0 %)	43	22 (51.2 %)	0.01
HbA1c at presentation, %	1691	11.5 (2.2)	1446	11.5 (2.2)	83	12.1 (2.1)	39	12.0 (2.4)	0.03
Interpreter use	1832	146 (8.0 %)	1570	49 (3.1 %)	88	35 (39.8 %)	43	29 (67.4 %)	<0.001
Tailored diabetes education^c^	1701	192 (11.3 %)	1570	103 (6.6 %)	88	53 (60.2 %)	43	36 (83.7 %)	<0.001
Length of stay, days^d^	1806	2.15 (1.08)	1545	2.12 (0.97)	88	3.14 (2.60)	43	3.99 (2.04)	<0.001

a Comparison across health literacy groups.

b Other insurance includes international or unavailable.

c Documented for patients with health literacy assessment.

d Data shown as median (IQR).

DKA, Diabetic ketoacidosis.

**Table 2:** Multivariable association of caregiver health literacy with clinical and diabetes management characteristics (n = 1693).

Characteristic	Possibility of Limited vs Adequate Literacy	High Likelihood of Limited vs Adequate Literacy
	Adjusted^b^ OR (95 % CI) for Characteristic	Adjusted^b^ OR (95 % CI) for Characteristic
Interpreter use	13.22 (7.66, 22.81)^a^	33.21 (15.99, 68.94)^a^
Tailored diabetes education	15.98 (9.79, 26.09)^a^	46.31 (19.72, 108.76)^a^
DKA on presentation	1.45 (0.93, 2.27)	1.43 (0.76, 2.67)

DKA, diabetic ketoacidosis. OR, odds ratio. CI, confidence interval.

a P<0.001.

b Models adjusted for sex, patient age, and insurance (public and private only).

**Table 3:** Association of caregiver health literacy and use of diabetes technology within first year (n=1091).

Diabetes Technology	Possibility of Limited vs Adequate Literacy			High Likelihood of Limited vs Adequate Literacy		
	Unadjusted OR (95 % CI)	Adjusted^b^ OR (95 % CI)	Adjusted^b^ OR (95 % CI) 2019–2022	Unadjusted OR (95 % CI)	Adjusted^b^ OR (95 % CI)	Adjusted^b^ OR (95 % CI) 2019–2022
CGM	0.79 (0.42, 1.51)	1.06 (0.44, 2.57)	1.0 (0.35, 2.84)	0.31 (0.11, 0.86)^a^	0.25 (0.06, 0.94)^a^	0.26 (0.07, 0.98)^a^
Insulin pump	0.18 (0.02 1.30)	0.23 (0.03 1.86)	0.23 (0.03, 1.92)	0.35 (0.05, 2.66)	0.84 (0.09, 7.49)	0.88 (0.10, 8.00)

CGM, continuous glucose monitor. OR, odds ratio. CI, confidence interval. Adjusted n=1081; Adjusted 2019–2022 n=628.

a P<0.05.

b Models adjusted for sex, patient age, insurance (public and private only), DKA, diagnosis year, length of stay, and use of tailored diabetes education.
