# Supplementary material for: Association between caregiver health literacy and patient characteristics in pediatric new onset diabetes mellitus
Source: Public Health. Author manuscript; Available in PMC 2025 Dec 11. (PMC12696676; doi:10.1016/j.puhe.2025.105892)

Supplemental Figure 1. Example Tailored Diabetes Education Sheets

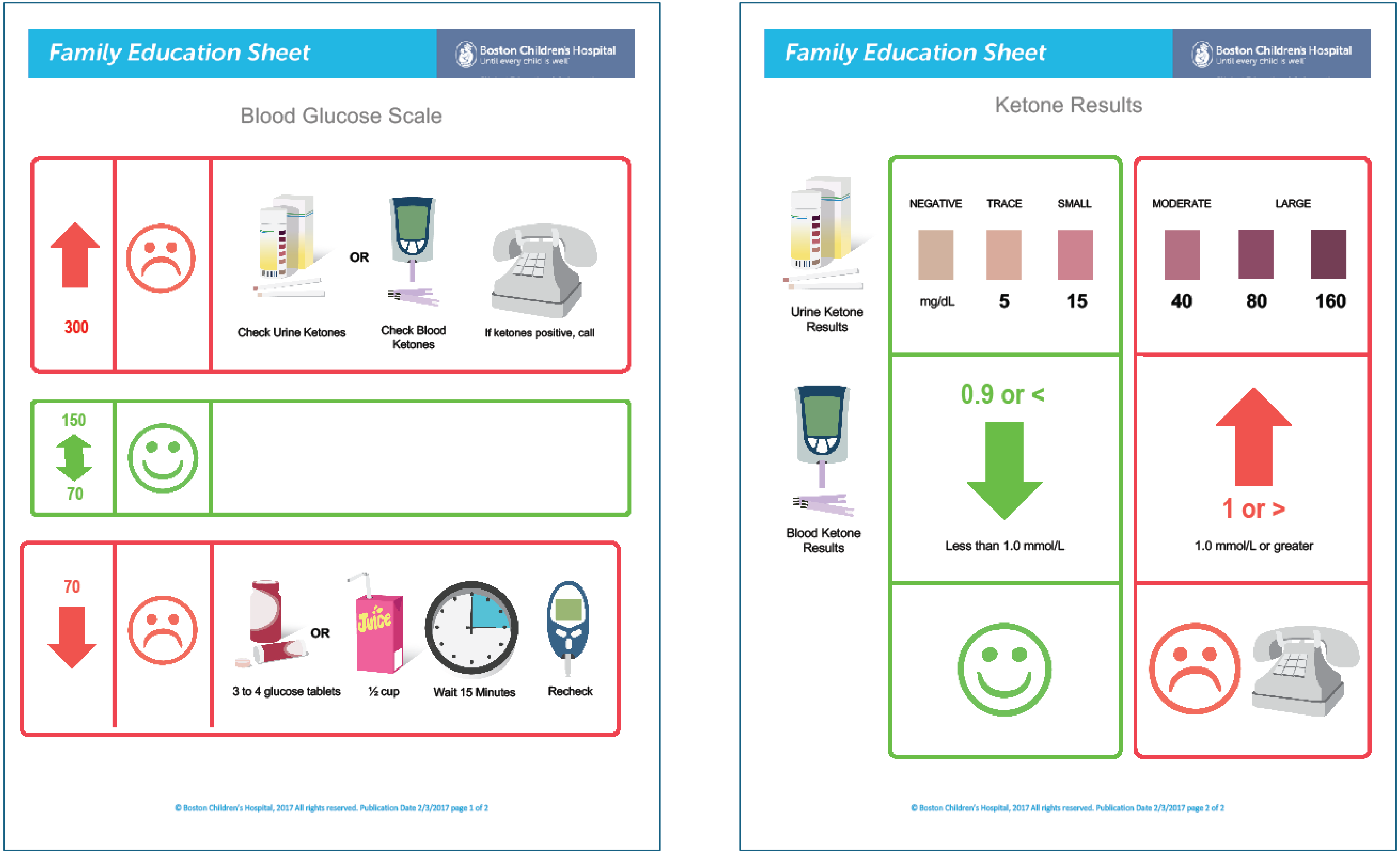

Supplement: MMC1 [file NIHMS2104134-supplement-MMC1.pdf]
